# Objectively measured sedentary behavior and physical activity in a sample of Finnish adults: a cross-sectional study

**DOI:** 10.1186/s12889-016-3591-y

**Published:** 2016-09-01

**Authors:** Pauliina Husu, Jaana Suni, Henri Vähä-Ypyä, Harri Sievänen, Kari Tokola, Heli Valkeinen, Tomi Mäki-Opas, Tommi Vasankari

**Affiliations:** 1 The UKK Institute for Health Promotion Research, Tampere, Finland; 2 Department of Welfare, The National Institute for Health and Welfare (THL), Helsinki, Finland; 3 Department of Health, The National Institute for Health and Welfare (THL), Helsinki, Finland

**Keywords:** Physical activity, Sedentary behavior, Accelerometer, Adults

## Abstract

**Background:**

Regular physical activity (PA) confers many positive effects on health and well-being. Sedentary behavior (SB), in turn, is a risk factor for health, regardless of the level of moderate to vigorous PA. The present study describes the levels of objectively measured SB, breaks in SB, standing still and PA among Finnish adults.

**Methods:**

This cross-sectional analysis is based on the sub-sample of the population-based Health 2011 Study of Finnish adults. The study population consisted of 18-to-85-year old men and women who wore a waist-worn triaxial accelerometer (Hookie AM 20) for at least 4 days, for at least 10 h per day (*n* = 1587) during a week. PA and SB were objectively assessed from the raw accelerometric data using novel processing and analysis algorithms with mean amplitude deviation as the processing method. The data was statistically analyzed using cross-tabulations, analysis of variance and analysis of covariance.

**Results:**

The participants were on average 52 years old, 57 % being women. Participants were sedentary 59 % of their waking wear time, mainly sitting. They spent 17 % of the time standing still, 15 % in light intensity PA, 9 % in moderate PA and less than 1 % in vigorous PA. Participants aged 30–39 years had the highest number of breaks in SB per day. Younger participants (<30 years of age) had more moderate and vigorous PA than older ones (≥60 years of age), and 30–60-year-olds had the greatest amount of light PA.

**Conclusions:**

Participants spent nearly 60 % of their waking time sedentary, and the majority of their daily PA was light. From a public health perspective it is important to find effective ways to decrease SB as well as to increase the level of PA. Our analysis method of raw accelerometer data may allow more precise assessment of dose-response relationships between objectively measured PA and SB and various indicators of health and well-being.

## Background

Physical activity (PA) confers many positive effects on health and well-being [1]. Regular PA can be a safe and low-cost medicine for several health problems, and importantly, be an effective means to prevent these problems and related disability. Sedentary behavior (SB) is a distinct behavior from moderate to vigorous PA (MVPA) [2]. By definition, SB means any waking behavior characterized by an energy expenditure ≤1.5 METs in a sitting or reclining posture [3]. Thus, SB is a separate construct from physical inactivity, which indicates low levels of PA, not reaching the level of the current recommendation for health-enhancing PA [3]. SB covers several facets, for example purpose, environment, type, posture, social and time, all of which include several sub-categories, for example purpose can cover work, education, transport, eating, rest and leisure [4]. Several studies have indicated that SB is a risk factor for cardiorespiratory and metabolic health [5–8], musculoskeletal health [9], depression [10] and mortality [1, 11], regardless of the level of MVPA [12], but not all studies confirm this independency [13].

Measurement of both SB and PA is important for describing the prevalence of these behaviors in different populations, in determining secular trends in these behaviors, for evaluating effects of interventions and for determining dose-response influence on specific health outcomes [14]. Traditionally the knowledge on SB and PA has largely rested on self-reports [15] whose validity and reliability is quite poor [16–19].

Technological development has enabled measuring new aspects of PA and SB [20, 21]. New devices have made it possible to record volume, duration, intensity and frequency of activities [22]. These new tools also offer a possibility to categorize individuals more specifically according to their PA and SB levels, which may be useful in targeting health promotion actions more precisely. For example, accelerometer data allows identification of individual PA patterns which may help to personalize PA counseling and goal setting However, criterion validity of these objective measurements varies a lot [23]. Most population studies describing objectively measured PA and SB have used count-based methods using varying analysis algorithms [24–29], which makes the direct comparison between studies very challenging. Thus, tri-axial accelerometer storing information as raw data instead of proprietary units (counts) has been proposed the method of choice when accurate and specific assessment of SB and PA is of primary importance [21, 30, 31]. Utilization of raw tri-axial acceleration data may advance comparisons between studies and different devices.

The purpose of the present study was to describe the levels of SB and PA in a sample of Finnish adults using raw data from tri-axial accelerometer which were analyzed with novel, validated analysis algorithms [30, 32]. More specifically, the study aimed at describing the amount of SB, number of breaks in SB, amount of standing still and the amount and intensity (light, moderate, vigorous) of PA. Furthermore, a novel classification scheme for different PA levels and number of steps is proposed. Combining several accelerometer parameters may help identifying individual activity patterns and thus facilitate targeted actions to reduce SB and promote PA more effectively.

## Methods

### Participants

The study is a part of a population-based Health 2011 Study [33], which is a multifactorial health examination study conducted with a stratified two-stage cluster sample of Finnish adults. Mainland Finland was divided into 20 strata defined by the 15 largest towns and the remaining rural areas based on the five university hospital regions. The sample represents the target population of each stratum, with exception of immigration after year 2000. The present study is based on the sub-sample (*n* = 4916) of the Health 2011 Study, of which 50 % (*n* = 2455) participated in the study. The data collection of the study was conducted between August 2011 and March 2012. Of those 2055 (84 %) were willing to take the waist-worn accelerometer (Hookie AM 20, Traxmeet Ltd, Espoo, Finland) for seven-day-measurements. The present study sample comprised of 18–85-year-old men and women (*n* = 2040) who used the accelerometer to sufficient extent; at least 4 days with a minimum of 10 h per day. Previous studies (e.g. [27, 34]) have used the similar criteria. In the present study 1587 persons met this criterion. All participants gave a signed informed consent before participation.

### Measurement of PA and SB

The accelerometer was attached to a flexible belt on the right hip and the participants were instructed to wear the belt for seven consecutive days during waking hours, except during showering and other water activities. The accelerometers were returned by mail to the research institute where the stored data were copied to a hard disk and analyzed later. The Hookie AM 20 device measures and stores the acceleration of the device in three orthogonal x, y and z directions at sampling rate of 100 Hz. The resultant acceleration (i.e. the magnitude of the acceleration vector) was determined from these three components. Then the mean amplitude deviation (MAD) of the resultant was analyzed in 6 s epoch length [30]. The MAD value describes the mean value of the dynamic acceleration component and it is independent of static gravity component. PA was categorised into three intensity categories based on metabolic equivalents (MET): light, moderate and vigorous. Classification has been validated with simultaneous measurements of MAD values and oxygen consumption (mean *r* = 0.958) during a non-stop pace-conducted walking and running test on an indoor track [32]. Light PA was defined as activity corresponding 1.5–2.9 MET, moderate activity as 3.0–5.9 MET and vigorous activity more than 6 MET [35]. Intensities of cycling and Nordic skiing were slightly underestimated by the accelerometer used and water-based activities were excluded.

Steps were identified by applying the method described by Ying et al. [36] and Mizell [37]. Instead of adaptive thresholds a fixed threshold was used. According to the definition of SB [3], time spent in sitting and reclining positions were combined to indicate SB, while standing still was analysed separately. It is possible to accurately determine whether the participant is standing, sitting or lying by applying the tri-axial information from the accelerometer. Since the body position during walking is upright and the direction of Earth’s gravity vector is constant, the vertical position (angle) of the accelerometer can be identified during normal walking. This known position can then be used for recognizing different body postures. In standardized conditions, standing can be separated from sitting or lying with 100 % accuracy, and sitting from lying with 95 % accuracy [38]. Daily amount of stand-ups (breaks in sedentary time) was calculated on the basis of the number of lying/sitting periods ending with a standing up. The standing up was detected if the MAD value was greater than 50 mg (milligravity) for the preceding or same epoch when the measured posture changed to standing. Participants whose daily measurement time was over 20 h were considered to have slept with the accelerometer. To avoid possible bias in SB time, their waking wear time was limited to 20 h and the exceeding time was reduced from the lying time.

#### PA classification

**Fig. 1:**
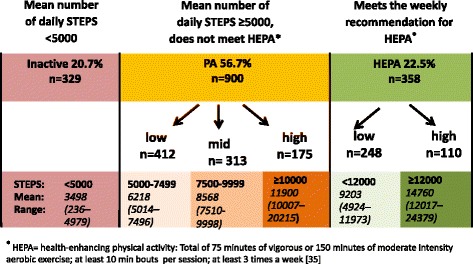
Pattern of physical activity. Six categories for individual physical activity patterns broken down by the number of daily steps and whether the person met the weekly recommendations for health-enhancing PA (HEPA)

The classification scheme of PA was formed on the basis of intensity and duration of PA in addition to the number of daily steps (Fig. 1). The classification was based on the 1 min moving average (mean of 10 last 6 s epochs) of the resultant acceleration. The participants meeting the current health-enhancing PA (HEPA) recommendation for aerobic activity (at least 150 min of moderate intensity or at least 75 min of vigorous intensity aerobic PA per week consisting of bouts lasting at least 10 min at the time or an equivalent combination of both intensities) [35] formed the HEPA-group. The HEPA-group was further divided in two sub-groups according to the number of mean daily steps. The step limits were based on the distribution of the present study sample utilizing the limits proposed by Tudor-Locke et al. [39]. Those taking less than 12000 steps per day formed HEPA-low group and those taking 12000 steps or more were named HEPA-high group. The participants not meeting the HEPA recommendation, but taking on average more than 5000 steps per day formed PA-group. This group was further broken down to three sub-groups: PA-low taking 5000–7499 steps per day, PA-mid taking 7500–9999 steps and PA-high taking 10000 or more steps per day. The participants taking on average less than 5000 steps per day [40] were regarded as inactive.

### Assessment of background characteristics

In order to describe the background characteristics of the present study sample the data collected by a Health 2011 Study -questionnaire was utilized. The participants were asked to report their educational background (no vocational education, vocational education, university degree) and marital status (cohabited, single). They also were asked to assess, how they perceived their health status and physical fitness (good, fairly good, poor), as well as the level of their PA lasting at least 10 min at the time separately for aerobic and musculoskeletal activities. This PA data was used to assess whether the participants met the current recommendation for HEPA [35]: 1) met the whole recommendation (moderate intensity aerobic PA for at least 150 min per week, vigorous intensity PA for at least 75 min per week or an equal amount of MVPA spread throughout the week and musculoskeletal activity at least twice a week), 2) met aerobic part, 3) met musculoskeletal part and 4) did not meet the recommendation.

### Statistical analysis

The data was analyzed by SPSS software, version 22 (SPSS Inc, Chicago IL) using descriptive methods (cross-tabulation, analysis of variance, analysis of covariance). The results are presented in age and sex specific groups. Because of skewed distributions, gamma regression was used to test the difference between age groups and sex on SB- and MVPA-bouts. Sex comparisons were adjusted for age and age group comparisons for sex. For multiple comparisons Sidak-adjustment was used. *P*-value less than 0.05 was considered as statistically significant.

## Results

The participants, who sufficiently wore the accelerometer for at least 4 days and at least during 10 h per day had on average higher education and better self-rated health and self-rated fitness than those who did not wear accelerometer or those who did not meet the criteria for sufficient wear time (Table 1). Most of the participants with sufficient accelerometer use had used it on six days during the 7-day-period. The mean waking wear time was 14 h 5 min per day, men had 19 min longer mean waking wear time than women (Table 2). The mean age of the participants was 52 years (SD 14.7) and 57 % of them were women. The mean weight was 84.9 kg (SD 14.6) for men and 70.7 kg (SD13.5) for women, and the mean height was 177.5 cm (SD 6.8) for men and 164.0 cm (SD 6.6) for women. The mean BMI was 26.9 (SD 4.2) for men and 26.3 (SD 4.8) for women. Thirty-eight percent of the participants had BMI <25.0 kg/m^2^, 38 % had BMI between 25.0 and 29.9 kg/m^2^ and 24 % had ≥30 kg/m^2^.

**Table 1:** Background characteristics of the participants aged 18–85 years

		Met the criteria^a^	Did not meet the criteria	Did not wear accelerometer	*p*-value^b^
		*n* = 1587	*n* = 263	*n* = 190	
Sex	Men, %	42.6	41.4	42.6	0.939
	Women, %	57.4	58.6	57.4	
Age group	18–29, %	5.3	5.0	3.7	0.264
	30–39, %	14.7	12.2	13.7	
	40–49, %	22.9	22.8	22.1	
	50–59, %	22.5	17.1	21.6	
	60–69, %	20.4	24.7	26.3	
	70–85, %	14.2	18.3	12.6	
Education	No vocational education, %	10.8	14.8	8.7	0.025
	Vocational education, %	62.1	66.4	66.8	
	University degree, %	27.1	18.8	24.5	
	Missing (n)	87	13	6	
Marital status	Cohabited, %	76.2	67.6	81.0	0.003
	Single, %	23.8	32.4	19.0	
	Missing (n)	87	13	6	
Perceived health	Good, %	47.7	38.8	42.9	0.022
	Fairly good/poor, %	52.3	61.2	57.1	
	Missing (n)	88	13	6	
Perceived fitness	Good, %	40.8	30.2	33.7	0.002
	Fairly good/poor, %	59.2	69.8	66.3	
	Missing (n)	94	15	6	
HEPA	Met the whole recommendation^c^, %	12.2	8.6	13.9	0.046
	Met aerobic part, %	28.3	22.0	28.9	
	Met musculoskeletal part, %	11.0	9.8	8.6	
	Did not meet the recommendation, %	48.6	59.6	48.7	
	Missing (n)	27	8	3	

^a^at least 4 days, at least 10 h/day

^b^Chi^2^-test

^c^met the whole recommendation: moderate intensity aerobic PA for at least 150 min per week, vigorous intensity PA for at least 75 min per week or an equal amount of MVPA spread throughout the week and musculoskeletal activity for at least twice a week [35]

**Table 2:** The proportions of days (%) with at least 10 h measurement-time and the mean accelerometer wearing time

		Age group					
		18–29	30–39	40–49	50–59	60–69	70–85
	Men	*n* = 26	*n* = 101	*n* = 144	*n* = 162	*n* = 153	*n* = 90
Number of days meeting the 10 h -criteria	4	3.8	13.9	13.2	13.0	10.5	14.4
	5	15.4	19.8	20.8	21.0	15.7	23.3
	6	61.5	45.5	45.8	47.5	54.2	53.3
	7	19.2	20.8	20.1	18.5	19.6	8.9
Wearing time	Mean	14 h 32 min	14 h 32 min	14 h 31 min	14 h 21 min	13 h 57 min	13 h 39 min
	SD	57 min	1 h 19 min	1 h 20 min	1 h 26 min	1 h 19 min	1 h 31 min
	Women	*n* = 58	*n* = 132	*n* = 219	*n* = 195	*n* = 171	*n* = 136
Number of days meeting the 10 h -criteria	4	12.1	7.6	9.1	7.7	9.9	9.6
	5	13.8	15.9	21.0	21.0	14.0	22.8
	6	55.2	48.5	53.0	56.4	60.8	52.9
	7	19.0	28.0	16.9	14.9	15.2	14.7
Wearing time	Mean	13 h 43 min	14 h 14 min	14 h 16 min	14 h 6 min	13 h 38 min	13 h 23 min
	SD	1 h 54 min	1 h 2 min	1 h 14 min	1 h 8 min	1 h 13 min	1 h 14 min

**Fig. 2:**
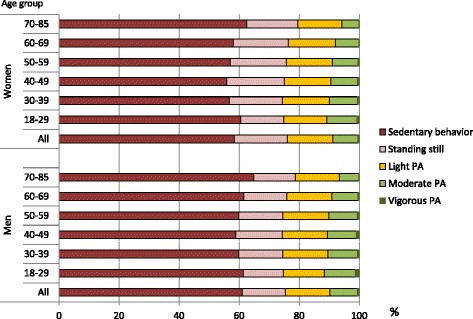
Proportions of sedentary behavior, standing still and physical activity. Proportions of sedentary behavior, standing still, and light, moderate and vigorous physical activity in relation to measurement time by age-group and sex

Irrespective of age and sex, the participants spent most of their waking hours sedentary. SB covered on average 59 % and standing still 17 % of the waking wear time. Participants spent 15 % of their waking hours in light PA. The mean proportion of MVPA was on average 9 % (moderate PA nearly 9 %, vigorous PA less than 1 %) (Fig. 2).

**Fig. 3:**
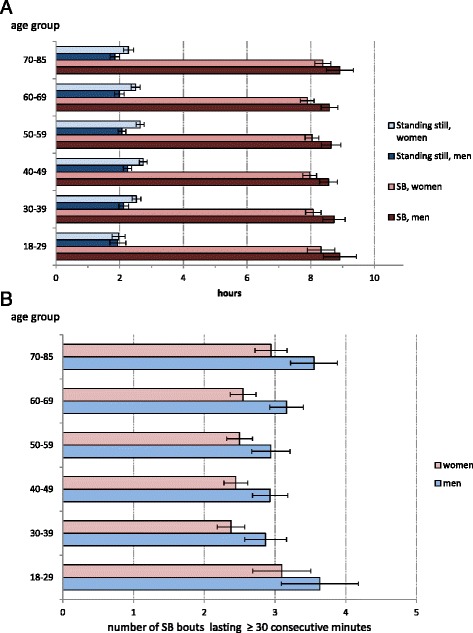
**a** Mean daily hours of sedentary behavior and standing still. Mean daily hours of sedentary behavior and standing still with 95 % confidence interval by age-group and sex. **b** Mean number of sedentary bouts lasting at least 30 consecutive minutes. Mean number of sedentary bouts lasting at least 30 consecutive minutes with 95 % confidence interval by sex

Figure 3a presents the mean daily hours of SB and standing still separately for the men and women. On average participants spent 8 h 20 min of their waking hours sedentary, in a sitting or reclining position and on average 2 h 20 min standing still. Men were on average more sedentary than women (8 h 40 min vs. 8 h 4 min, *p* < 0.001), while women were standing on average 27 min more per day than men (*p* < 0.001). When the breaks in SB were assessed as a mean number of standing-ups per day, the middle-aged participants broke the sedentary periods most often. The youngest group had on average 42 standing-ups per day, 30–39-year-olds had 51, 40–49-year-olds 48, 50–59-year-olds 47, 60–69-year-olds 43 and those 70-years of age or older had 36. Women had on average 3 more standing-ups per day than men (*p* < 0.05). Women had on average less long (≥ 30 min) sedentary bouts per day than the men (2.6 vs. 3.1, *p* < 0.001). Regarding the age-groups, the oldest (≥70 years) and the youngest (18–29 years) participants had on average more long sedentary bouts per day than the other participants (*p* < 0.05). The participants aged 30–39 years had on average the smallest amount of long sedentary bouts (Fig. 3b).

**Fig. 4:**
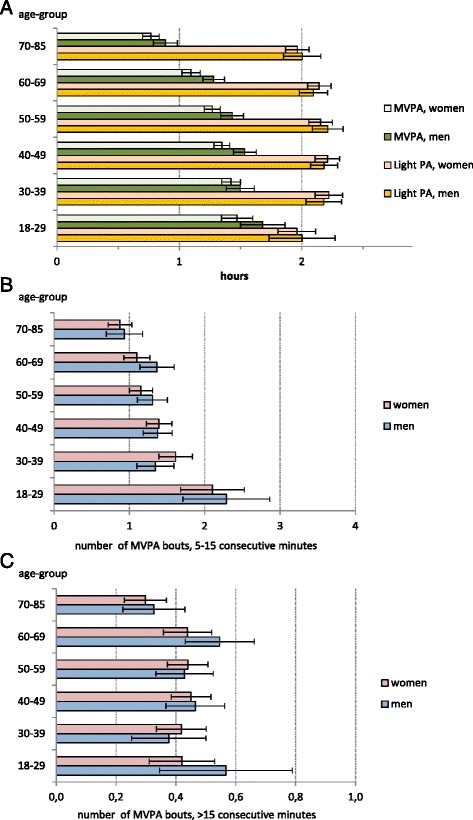
**a** Mean daily hours of physical activity. Mean daily hours of light and moderate-to-vigorous physical activity with 95 % confidence interval by age-group and sex. **b** Mean number of moderate-to-vigorous physical activity bouts of 5–15 consecutive minutes. Mean number of moderate-to-vigorous physical activity bouts lasting 5–15 consecutive minutes with 95 % confidence interval by sex. **c** Mean number of moderate-to-vigorous physical activity bouts over 15 consecutive minutes. Mean number of moderate-to-vigorous physical activity bouts lasting over 15 consecutive minutes with 95 % confidence interval by sex

Figure 4a presents the daily hours of light PA and MVPA. The mean time of light PA was slightly over 2 h (2 h 8 min) and that for MVPA 1 h 17 min per day. The 30–60-year-olds had on average more light PA than the youngest and the oldest participants, but the individual variation was large. In each age- and sex-group there were both very active and very inactive participants. The youngest participants had more MVPA than the older groups, but again individual variation was large, especially among the men aged under 30 years. Participants younger than 30 years took on average 9043 steps per day (95 % CI 8351–9736), while the participants aged over 70 years took on average 5183 steps (95 % CI 4762–5604). Figure 4b and 4c presents the mean number of MVPA bouts lasting 5–15 consecutive minutes (4b) and the number of bouts exceeding 15 consecutive minutes (4c). There were no statistically significant sex differences in these bouts. The oldest participants (≥70 years) had on average less MVPA bouts of 5–15 min than all the younger participants (*p* < 0.001 to *p* = 0.035). Regarding the longer bouts, the oldest participants differed only from the 60–69-year-olds (*p* = 0.005) and 40–49-year-olds (*p* = 0.020).

Regarding the proposed classification scheme of PA-patterns (see Fig. 1), over a half of the participants (57 %) belonged to PA-group, not meeting the HEPA recommendation, but taking on average over 5000 steps per day. Men and women were equally distributed into each group, but the mean age of the inactive-group was higher than that of the other groups (*p* < 0.001).

**Fig. 5:**
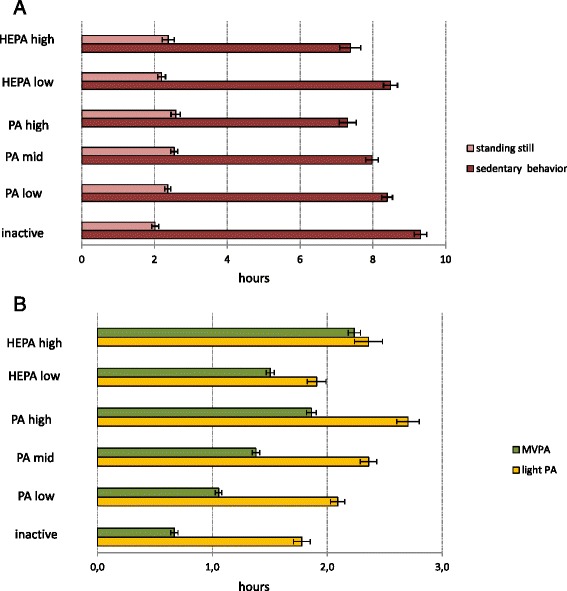
**a** Mean hours of sedentary behavior and standing still according to physical activity categorization. Mean hours of sedentary behavior and standing still in six categories of physical activity categorization with 95 % confidence interval, adjusted for age and sex. **b** Mean hours of physical activity according to physical activity categorization. Mean hours of light and moderate-to-vigorous physical activity in six PA-categories with 95 % confidence interval, adjusted for age and sex

Figure 5a and b present the age- and sex-adjusted SB, standing and PA times in the six PA categories. Highest average amount of sedentary time was recorded in the inactive-group (Fig. 5a) which also had higher number of long (≥ 30 min) sedentary bouts than the other groups (*p* < 0.001). PA-high and HEPA-high -groups had on average similar amount of SB (7 h 20 min), while HEPA-low group was on average more sedentary (8 h 30 min).

The HEPA-high -group spent on average more time in MVPA than the other groups (Fig. 5b). The HEPA-low-group, on the other hand, had on average less MVPA than the PA-high-group (*p* < 0.001), less light activity than all the PA-groups (*p* < 0.01) and less standing time than the PA-mid and–high-groups (*p* < 0.001). PA-high-group had on average the greatest amount of light PA and the least number of long (≥ 30 min) sedentary bouts, although the difference in the number of long sedentary bouts between HEPA-high and PA-high-groups was not statistically significant.

## Discussion

The present study measured objectively various features of SB and PA using raw tri-axial accelerometer data. According to the results participants spent on average 59 % of their waking hours sedentary and additional 17 % by standing still. Light activity covered 15 % and MVPA less than one tenth of the waking wear time. The proposed classification scheme for PA was generally able to detect logical differences between the accelerometer-measured features of SB and PA in the six proposed PA categories. In the future studies as well as in the practise of PA counselling the novel classification may help in identifying individual activity patterns and thus facilitate to target actions to reduce SB and promote PA more precisely in a personalized fashion. Consideration of steps in the PA categorization revealed clear differences between and within the traditionally used categories (inactive, active, HEPA).

Despite weaknesses in the criterion validity of previously reported count-based measurements [23], the average 59 % daily sedentary time in the present study sample is in line with previously reported values (55–62 %) [24–27, 29]. In terms of hours the proportion of SB corresponds on average to 7.3–9 h per day [24–27, 29]. The mean SB (8.3 h per day) identified in the present study is also well in line with the former objective findings, as is the finding that men spent, on average, more time sedentary than women [27, 41].

Recent studies have reported that short breaks in SB are beneficial for health [42, 43] and may be particularly important for cardio-metabolic health [7]. It has also been suggested that breaking up sedentary time regularly and replacing it by light intensity PA is more amenable to change than increasing the amount of MVPA [2]. In the present study people aged 30 to 60-years had on average more breaks in SB than the youngest and oldest age groups. Interestingly, and according to the aforementioned hypothesis, they also had the greatest time of light PA.

In the present study, the mean daily proportion of light PA was 15 % which is considerably less than the 39 % reported by Healy et al. [26] and Spittaels et al. [24] and the 34 % of low-intensity PA and lifestyle activity reported by Hansen et al. [27]. This is most likely due to differences in the representativeness of the study populations as well as differences in measurement methods and analysis algorithms. Most of the previous population studies have not differentiated standing from SB. However, it is well-agreed that standing is a separate behavior from SB [3, 44]. Some standing with slight movement or swaying might have been classified as light PA in the previous studies. The rationale to separate standing from light PA in the present study was that the energy expenditure of standing is lower than that of light PA [45], while despite the static nature of standing, as a prolonged continuous behavior it may lead to specific health problems [46].

The proportion of MVPA in the present study sample was on average 9 % of waking hours. The values are somewhat higher than those reported for adults in previous studies, from 4 % [26, 27, 29] to 6 % [24]. The comparison between studies is challenging since the analysis algorithms [21] as well as the cut-points for MVPA [14] have varied a lot between the studies.

The proposed classification scheme for individual PA-patterns was able to detect logical differences in parameters describing SB and PA between the groups. The inactive-group had the greatest amount of SB. Although the PA-high group did not meet the HEPA recommendation [35], the mean total time of MVPA in that group was greater than that in the HEPA-low -group. PA-high -group had also the greatest amount of light PA. For public health reasons, on a population level, it is important to promote total PA and to decrease SB. These two targets need different tools and actions and the proposed PA-patterns may help to target these actions appropriately. For example the proposed PA-pattern indicates that being active enough to meet HEPA recommendation, as in the HEPA-low-group, may simultaneously mean that the total amount of PA is lower and the amount of SB is higher than in the PA-high group not meeting the recommendation. Thus, replacing SB by light PA might be the most important target in HEPA-low group. In the PA-high -group, in turn, promotion of longer MVPA-bouts to meet the HEPA recommendation could be a way to increase health benefits of PA while keeping the previous level of light activity. The inactive group needs more PA at any intensity while the amount of SB needs to be reduced. The most feasible way of doing that could be adding active breaks into sedentary time and promoting light PA in terms of active commuting and everyday activities. Thus, the proposed categorization offers a more specific and more personalized tool to identify PA-patterns than the traditionally used categorization based on HEPA-criteria only.

### Strengths and limitations

The strength of the present study is that several features of PA and SB were measured with a tri-axial accelerometer collecting data in raw mode as recommended [21, 31]. The data was analyzed with novel, universal and valid algorithms [30, 32] and the study sample included both men and women within a wide age range. Although the accelerometer used in the present study is not widely used, the analysis methods are universal and can be used with any tri-axial accelerometer collecting data in raw mode [30]. Weaknesses of the study were that the currently used analysis algorithms may not accurately recognize movements performed only with lower-or upper-extremities (eg. gym exercises) and movements performed in supine position (eg. pilates). Also the intensities of cycling and Nordic skiing are not adequately captured at the moment [22]. Further, accelerometer used in the study was not water-resistant which means that water-activities, like swimming, were not included. The cross-sectional design of the study is also a limitation to causal interpretation of the results and trend analysis. The Health 2011 nonparticipation and representativeness has been presented in more detail elsewhere [47]: in general, the participation to Health 2011 sample was acceptable 72,5 % but lower participation was observed among young age-groups, men and among those with low educational attainment. Moreover, the overall participation rate of 50 % from the original sample, limits the generalization of the results to the adult Finnish population. Also, unequal participation of the age- and sex groups limits the generalizability of the results.

## Conclusions

Finnish adults in this study were sedentary almost 60 % of the waking hours, and the majority of daily PA was light. From a public health perspective it is important to find effective ways to decrease SB as well as to increase the level of daily PA. A proposed classification scheme based on the validated analysis of the tri-axial acceleration data and number of daily steps may help in personalized targeting and planning PA promotion policies appropriately.

In the future it is important to assess objectively measured SB and PA in representative study populations in order to get representative and reliable data on the level of these behaviors in the population. Furthermore, the dose-response relationships between objectively measured PA and SB and various indicators of health and well-being, for example lipid- and glucose metabolism [42], coronary heart diseases [48] and low back pain [49], need to be studied. Different PA and SB bouts over day need to be studied in more detail.

Especially the health effects of objectively measured light, sporadic activity and on the other hand effects of different bouts of PA and SB and breaks in SB on health outcomes need to be elaborated.

## Abbreviations

CI: Confidence interval
HEPA: Health-enhancing physical activity
MET: Metabolic equivalent
MVPA: Moderate-to-vigorous physical activity
PA: Physical activity
SB: Sedentary behavior
SD: Standard deviation
